# Association of Depression With 10-Year and Lifetime Cardiovascular Disease Risk Among US Adults, National Health and Nutrition Examination Survey, 2005–2018

**DOI:** 10.5888/pcd19.210418

**Published:** 2022-05-26

**Authors:** Steven D. Barger, Gabrielle C. Struve

**Affiliations:** 1 Department of Psychological Sciences, Northern Arizona University, Flagstaff, Arizona

## Abstract

**Introduction:**

Although an association between depression and incident cardiovascular disease (CVD) risk has been established, no US studies have quantified this association using standard primary care assessments or among younger adults who are not routinely screened for CVD risk. We estimated the association of mild and major depression with 1) 10-year atherosclerotic CVD (ASCVD) risk for people aged 40 to 79 years and 2) high lifetime CVD risk prevalence for people aged 20 to 39 years.

**Methods:**

We conducted a cross-sectional analysis of data from the 2005–2018 National Health and Nutrition Examination Survey for adults aged 20 to 39 years (n = 10,588) and adults aged 40 to 79 years (n = 16,848). We used the Patient Health Questionnaire-9 [PHQ-9] to classify no depression (PHQ-9 score, 0–4), mild depression (PHQ-9 score, 5–9) and major depression (PHQ-9 score ≥10).

**Results:**

Among women aged 40 to 79, ASCVD absolute risk was 6.0% for no depression, 6.9% for mild depression, and 7.6% for major depression (*P* < .001 vs no depression). Among men aged 40 to 79, the corresponding absolute ASCVD risks were 9.9%, 11.1%, and 11.3%, respectively (*P* < .001 vs no depression). High lifetime CVD risk prevalence for women aged 20 to 39 was 41.9% for no depression, 53.2% for mild depression, and 66.5% for major depression (*P* < .001 vs no depression). For men aged 20–39 the corresponding high lifetime risk percentages were 53.3%, 64.8%, and 74.4% respectively (*P* < .001 vs no depression).

**Conclusion:**

Mild and major depression are associated with elevated 10-year ASCVD risk and substantially elevated lifetime CVD risk among younger people ineligible for ASCVD risk assessment. Jointly addressing depression and CVD risk and extending prevention efforts to younger adults are warranted.

**Summary**

**What is already known on this topic?**

Depression is associated with elevated cardiovascular disease (CVD) risk; however, this association has not been characterized using contemporary US primary prevention assessment standards for depression and CVD.

**What is added by this report?**

Our study shows greater CVD risk for both clinical and subclinical depression, including higher lifetime CVD risk among adults aged 20 to 39 years.

**What are the implications for public health practice?**

The association of depression with CVD is consistent, and the gradient is stronger for lifetime versus 10-year risk. Depression and CVD risk are prevalent, modifiable conditions that warrant population-level intervention.

## Introduction

Unipolar depression is observed in 7% of the US adult population (1), is a leading cause of disability (2), and in 2018 was noted in more than 10 million office visits in the US (3). Depression is also an established marker of incident cardiovascular disease (CVD) risk (4,5), the leading cause of death worldwide. Together, depression and CVD are associated with premature death, and both are principal contributors to the rise in disability-adjusted life years observed from 1990 to 2019 (2). Alleviating depression and reducing CVD risk are therefore paramount for public health, and the prevalence and comorbidity of these conditions underscore the importance of considering them together. This consideration can be facilitated by characterizing the association of depression with CVD risk while risk modification is possible (ie, among people free of clinical CVD).

Although primary care depression screening is recommended in the US (6), heterogeneous depression screening assessments are a barrier to reliably characterizing the magnitude of the depression–CVD risk association and to establishing a harmonized clinical and public health infrastructure to treat and prevent these conditions (7). Many different depression assessments exist, and these differences create uncertainty regarding the association of depression with CVD risk. For example, some popular depression assessments do not align with clinical diagnostic criteria (eg, loneliness is an item in the Center for Epidemiological Studies Depression scale). Standardized, validated assessments, particularly those widely used in research and practice (8), reduce uncertainty and permit harmonization of depression assessments in patient registries and clinical care (7,9). The recommended depression assessment standard is the Patient Health Questionnaire (PHQ-9) (10). The PHQ-9 is brief, is self-administered, has excellent congruence with diagnostic interviews, and is widely used in clinical settings and in public health surveillance. Similarly, the pooled cohort equations (11) represent the current US standard for assessing 10-year atherosclerotic cardiovascular disease (ASCVD) risk among people aged 40 to 79 years who are free of clinical CVD (11). Describing the depression–CVD association using these 2 assessment standards can provide risk benchmarks relevant for both public health and clinical practice. To date, no studies have quantified the association between depression and CVD risk using these 2 US assessment standards. The first objective of this study was to estimate the association of 10-year ASCVD risk with PHQ-9 depression in a probability sample of US adults aged 40 to 79 years.

Adults aged 20 to 39 years represent 27% of the US population (12) and have a substantial prevalence of depression (13). However, because of the low risk of a clinical CVD event in people aged 20 to 39, there is no 10-year ASCVD risk algorithm for this group (11). However, lifetime CVD risk can be assessed among people aged 20 to 39 and can therefore provide a valid index of CVD risk at different levels of depression for adults otherwise overlooked by ASCVD-based primary prevention (14). The second objective of the study was to estimate the association between depression and high lifetime risk of CVD death for adults aged 20 to 39. Because depression below clinical thresholds is prognostic for incident CVD (5), we compared CVD risk for both mild and major depression relative to people without depression. Consistent with similar work (15), we hypothesized that mild and moderate depression are associated with higher 10-year and lifetime CVD risk relative to no depression.

## Methods

### Study design and sampling

We combined 7 consecutive cycles of the National Health and Nutrition Examination Surveys (NHANES; 2005–2018) to estimate the association of 10-year ASCVD and high lifetime CVD risk by depression. The PHQ-9 was not assessed before 2005. NHANES is a series of nationally representative cross-sectional samples of US residents. It includes a computer-assisted interview component assessing sociodemographic characteristics, smoking status, prescription medication use, and history of clinical CVD and depression (16). Participants who reported using prescription medication during the last 30 days were asked to show their medication(s) to NHANES staff. Staff entered the medications into a computer where they were matched to a prescription drug database. Blood pressure and blood samples to ascertain lipids and glycated hemoglobin A1c were collected during a separate session at a mobile examination center. The cholesterol assay changed after the 2005–2006 survey cycle, but as per NHANES documentation, no adjustments were necessary for the instrumentation change. Response rates for participants aged 20 to 79 ranged from 49% to 75% (median, 73.8%) for the interview and from 46% to 74% (median, 71.2%) for the examination. Participants provided informed consent, and the study protocol was approved by the National Center for Health Statistics Ethical Review Board.

Depression was assessed with the PHQ-9 (10). We divided subclinical depression into none/minimal depression (PHQ-9 score, 0–4; hereafter referred to as “not depressed”) and mild depression (PHQ-9 score, 5–9) (10). Clinical depression, defined as a score of 10 or higher, was labeled moderate-to-severe depression (10). This cut score has good sensitivity and specificity with structured clinical interview diagnoses of major depressive disorder (17), so hereafter we refer to this category as “major depression.”

### Risk scores

**Ten-year ASCVD risk.** Ten-year ASCVD risk scores were calculated according to previously published guidelines (11). Scores incorporate sex- and race-specific algorithms to predict 10-year absolute ASCVD risk. Risk scores have good classification accuracy, and unlike earlier risk prediction algorithms, model development included a substantial number of Black participants (11). Risk estimates are based on age, medicated or unmedicated systolic blood pressure, total cholesterol, high-density lipoprotein (HDL) cholesterol, diabetes status, and smoking status. Risk scores predict first occurrence of fatal or nonfatal stroke, nonfatal myocardial infarction, or coronary heart disease death. People aged 40 to 79 free of diagnosed CVD are eligible for ASCVD risk score calculation.

**High lifetime CVD risk.** Lifetime risk of death from CVD was defined by using 5 mutually exclusive categories of risk factors, which were calculated on the basis of systolic and diastolic blood pressure, total cholesterol, smoking status, diabetes status, and medication use for blood pressure or cholesterol (14). Categories are all risk factors optimal; 1 or more risk factors not optimal; 1 or more risk factors elevated; 1 major risk factor; and 2 or more major risk factors. Optimal risk is defined by unmedicated systolic and diastolic blood pressure values less than 120 and 80 mm Hg, respectively; unmedicated total cholesterol less than 180 mg/dL; being a nonsmoker; and being free of diabetes. Nonoptimal risk is the same as optimal except for systolic values of 120 to 139 mm Hg or diastolic values of 80 to 89 mm Hg; or total cholesterol 180 to 199 mg/dL. Elevated risk is defined by yet higher blood pressure (systolic, 140–159 mm Hg or diastolic, 90–99 mm Hg) or higher cholesterol (200–239 mg/dL). The remaining 2 risk categories are defined by the presence of major CVD risk factors, defined by high blood pressure (≥160 mm Hg systolic or ≥100 mm Hg diastolic; or antihypertensive medication use); high total cholesterol (240 mg/dL or taking lipid-lowering medications), being a current smoker, or having diabetes. Diabetes was defined as hemoglobin A_1c_ (HbA_1c_) at or above 6.5% or reported physician diagnosis of diabetes, or current use of insulin or oral hypoglycemic medications (18,19). We classified people aged 20 to 39 as at either low lifetime risk of CVD death (<39%; combining optimal with ≥1 not optimal risk categories) or high lifetime risk of CVD death (≥39%; combining the other 3 categories). This binary classification is based on a natural separation in lifetime death risk observed between optimal/not optimal versus higher risk categories (20), and this separation was consistently observed across birth cohorts, sex, and race (14). No health exclusions apply to lifetime CVD risk classifications, and unlike ASCVD risk scores, lifetime risk is not sensitive to age and is valid for people aged 20 to 39 as well as those aged 40 to 79 (14). One percent (95% CI, 1%–1%) of people aged 20 to 39 years reported prior CVD.

### Analysis

We performed regression analyses of ASCVD risk on 2 levels of depression, mild and major, using not depressed as the reference group. Because ASCVD risk scores (20) and depression (1) vary substantially by sex, we analyzed men and women separately. To minimize the influence of age on ASCVD risk scores, we additionally stratified analyses by 10-year age groups and included continuous age, centered at the subgroup mean, as a covariate. Cell sizes were too small for reliable estimation when further stratifying by race and ethnicity. Model fit was evaluated by testing whether a squared predicted score added significant prediction relative to the model with depression and age. Adequate model specification was achieved by including age and age squared, mean centered within each age/sex stratum. An additional cubic age term was necessary for adequate specification for sex-specific models with participants aged 40 to 79. We report sensitivity analyses excluding people with high LDL (>190 mg/dL) and high systolic blood pressure (>200 mm Hg) (11), adjusting for past-month prescription antidepressant use (21) and adjusting for statin use (22). Prescription antidepressant use was coded by using generic antidepressant names (21), and statin use was identified by the therapeutic category HMG-CoA reductase inhibitors. In a final sensitivity analysis, we adjusted for education and race and ethnicity.

To evaluate the association between depression and high lifetime risk, we used generalized linear models with a Poisson distribution and a log link with robust SEs to generate prevalence ratios (23) for mild and major depression relative to people without symptoms of depression.

All statistical analyses were conducted by using Stata software, version 16.1 (StataCorp LLC) and incorporated clustering, stratification, and weights to accommodate the complex survey design. Significance was determined by a 2-sided test with *P* ≤ .05. Each 2-year survey cycle weight was divided by the number of cycles to produce a sum of weights roughly equal to the population at the midpoint of the 2005–2018 interval (24). Estimates represent the civilian, noninstitutionalized US population for the respective age/sex groups and depression categories, with the ASCVD analyses generalizing to participants aged 40 to 79 who were free of existing CVD. We evaluated secular trends (24) in the depression–CVD risk association using an interaction term of depression with survey cycle year in sex-specific analyses. No interactions were observed for high lifetime CVD risk. For ASCVD risk, the interaction term for mild depression and survey cycle was significant for women aged 40 to 79, indicating increasing absolute risk for mild depression relative to no depression over the cycle years. When examining ASCVD risk in sex-specific 10-year age groups, there were 2 other significant interactions among 16 interaction terms. However, none of these depression coefficients changed when adding cycle year to ASCVD regression models, so we did not consider cycle year further (ie, analyses are based on the combined 14 years of NHANES data).

## Results

The not depressed group was composed predominantly of people reporting 1 or fewer PHQ-9 symptoms (65%) (Table 1). Major depression prevalence ranged from 6% to 10% for women and 3% to 6% for men. Subclinical depression prevalence was higher; 15% to 18% of women and 9% to 14% of men had mild depression. For women and men aged 20 to 39 years, mild depression was 16.8% and 13.9% and major depression was 9.7% and 5.4%, respectively.

**Table 1:** Participant Characteristics, by Depression Status, National Health and Nutrition Examination Survey, 2005–2018

Characteristic	Total	Depression^a^		
		Not depressed	Mild	Major
**Aged 40–79 y (10-year ASCVD risk sample)**				
Total no. of participants	16,848	12,887	2,560	1,401
Age, mean (SD), y	55.4 (8.6)	55.7 (8.5)	54.7 (8.6)	53.9 (8.4)
Women, % (no.)	52.6 (8,757)	50.0 (6,286)	60.8 (1,560)	64.5 (911)
Race and ethnicity, % (no.)				
Mexican American	6.8 (2,726)	6.6 (2,057)	7.2 (428)	8.1 (241)
Other Hispanic	4.7 (1,772)	4.3 (1,285)	5.1 (277)	7.7 (210)
White (non-Hispanic)	72.5 (7,056)	73.3 (5,405)	71.6 (1,080)	65.7 (571)
Black (non-Hispanic)	9.6 (3,586)	9.2 (2,733)	10.8 (568)	11.3 (285)
Other race	6.4 (1,708)	6.6 (1,407)	5.4 (207)	7.2 (94)
Education level, % (no.)				
<High school diploma	14.4 (4,195)	12.7 (2,939)	18.1 (738)	25.5 (518)
High school diploma	23.3 (3,833)	22.5 (2,882)	26.1 (630)	26.1 (321)
Some college	30.2 (4,697)	29.6 (3,570)	32.4 (732)	31.4 (395)
College graduate or higher	32.0 (4,111)	35.1 (3,488)	23.3 (457)	17.0 (166)
Missing	0 (12)	0 (8)	0.1 (3)	0.1 (1)
Mean (SD) systolic blood pressure, mm Hg	125.0 (14.5)	125.1 (14.2)	125.1 (15.1)	124.2 (16.8)
Antihypertensive medication, % (no.)	29.5 (5,595)	28.5 (4,128)	30.9 (916)	37.5 (551)
Total cholesterol, mean (SD), mg/dL	202.8 (34.9)	202.2 (34.1)	205.8 (36.5)	203.5 (40.6)
High density cholesterol, mean (SD), mg/dL	54.8 (14.6)	55.1 (14.5)	54.1 (14.9)	52.6 (14.7)
Diabetes, % (no.)	14.5 (3,352)	13.3 (2,360)	17.7 (616)	20.2 (376)
Current smoker, % (no.)	18.2 (3,245)	15.4 (2,141)	24.6 (621)	35.8 (483)
Statin medication, % (no.)	12.0 (2,114)	12.2 (1,653)	10.7 (295)	12.3 (166)
Antidepressant medication, % (no.)	13.2 (1,728)	9.4 (848)	22.3 (450)	35.9 (430)
**Aged 20–39 y (high lifetime CVD risk sample)**				
Total no. of participants	10,588	8,028	1,693	867
Age, mean (SD), y	29.4 (4.7)	29.4 (4.7)	28.8 (4.9)	29.6 (5.1)
Women, % (no.)	49.2 (5,407)	47.0 (3,898)	53.8 (957)	62.7 (552)
Race and ethnicity, % (no.)				
Mexican American	12.4 (1,981)	12.7 (1,556)	12.4 (306)	9.0 (119)
Other Hispanic	7.3 (1,000)	7.0 (728)	7.3 (166)	9.3 (106)
White (non-Hispanic)	59.8 (4,147)	60.2 (3,124)	58.6 (668)	58.0 (355)
Black (non-Hispanic)	11.9 (2,121)	11.4 (1,576)	12.3 (336)	15.9 (209)
Other race	8.7 (1,339)	8.7 (1,044)	9.4 (217)	7.8 (78)
Education level, % (no.)				
<High school diploma	14.5 (2,079)	13.3 (1,460)	16.6 (366)	21.8 (253)
High school diploma	22.1 (2,393)	21.2 (1,758)	24.9 (414)	26.4 (221)
Some college	34.8 (3,612)	33.6 (2,681)	38.8 (630)	38.9 (301)
College graduate or higher	28.6 (2,501)	31.9 (2,126)	19.7 (283)	12.9 (92)
Missing	0.1 (3)	0.1 (3)	0	0
**Cardiovascular risk factors**				
Mean (SD) systolic blood pressure, mm Hg	115.1 (9.9)	115.0 (9.8)	115.1 (10.1)	115.3 (11.2)
Antihypertensive medication, % (no.)	3.6 (395)	3.0 (246)	4.2 (75)	7.8 (74)
Total cholesterol, mean (SD), mg/dL	184.4 (31.2)	184.0 (30.2)	185.6 (34.4)	185.8 (34.7)
High density cholesterol, mean (SD), mg/dL	51.9 (12.2)	52.1 (12.1)	52.2 (12.4)	50.2 (12.2)
Diabetes, % (no.)	3.0 (381)	2.7 (256)	3.3 (71)	5.7 (54)
Current smoker, % (no.)	24.2 (2,605)	20.5 (1,675)	32.2 (545)	46.2 (385)
Statin medication, % (no.)	0.8 (76)	0.8 (55)	0.8 (15)	0.6 (6)
Antidepressant medication, % (no.)	6.2 (521)	3.3 (210)	12.7 (154)	22.3 (157)

Abbreviations: ASCVD, atherosclerotic cardiovascular disease; CVD, cardiovascular disease.

a Depression was measured using the Patient Health Questionnaire-9 (PHQ-9) and categorized as not depressed (score, 0–4), mild depression (score, 5–9), and major depression (score ≥10). Participants aged 40 to 79 years were included if they were free of clinical cardiovascular disease. Diabetes was defined as glycated hemoglobin ≥6.5, physician diagnosis of diabetes, or use of insulin or oral hypoglycemic medications (19).

### ASCVD risk

Ten-year ASCVD risk for women and men by depression category, adjusted for age, is presented in Table 2. Among men and women aged 40 to 79, both mild and major depression were associated with higher ASCVD risk relative to the not depressed group. A linear trend for this age range was observed for women (*P* = .002) but not men (*P* = .70) (Table 3). Within 10-year age groups, both mild and major depression were associated with higher 10-year risk for women, with the exception of mild depression for women aged 70 to 79 (Table 3). Both mild and major depression were associated with higher 10-year risk for men aged 40 to 49 and 50 to 59. Higher ASCVD risk was observed for mild but not major depression among men aged 60 to 69. Ten-year risk scores were not applicable for people with very high LDL or systolic blood pressure. Excluding these participants did not substantially change the estimates, nor did controlling for antidepressant use or statin use (estimates provided in Supplemental Table 1, available at https://osf.io/wcdxv/). Additional analyses excluding people with cancer diagnoses (excluding nonmelanoma skin cancer) were similar (data not shown). When adjusting for education and race and ethnicity, the associations were attenuated but persisted, except for the mild depression ASCVD coefficients for men aged 40 to 49 and 60 to 69 (estimates provided in Supplemental Table 2, available at https://osf.io/wcdxv/).

**Table 2:** Age-Adjusted Absolute 10-Year ASCVD Risk (%), by Depression, Stratified by Sex and Age, National Health and Nutrition Examination Survey, 2005–2018

Characteristic	Depression^a^		
	Not depressed	Mild	Major
**Women**			
**All (aged 40–79 y)**			
ASCVD risk (95% CI)	6.0 (5.7–6.2)	6.9 (6.6–7.2)	7.6 (7.2–8.0)
No.	6,286	1,560	911
**Aged 40–49 y**			
ASCVD risk (95% CI)	1.7 (1.6–1.8)	2.1 (1.8–2.3)	2.6 (2.3–3.0)
No.	1,871	480	305
**Aged 50–59 y**			
ASCVD risk (95% CI)	3.2 (3.0–3.4)	4.3 (3.8–4.8)	4.6 (4.2–5.1)
No.	1,625	439	303
**Aged 60–69 y**			
ASCVD risk (95% CI)	8.0 (7.7–8.3)	9.5 (8.9–10.2)	10.7 (9.7–11.7)
No.	1,788	422	224
**Aged 70–79 y**			
ASCVD risk (95% CI)	20.3 (19.7–20.8)	20.9 (20.0–21.9)	23.4 (22.0–24.9)
No.	1,002	219	79
**Men**			
**All (aged 40–79 y)**			
ASCVD risk (95% CI)	9.9 (9.6–10.2)	11.1 (10.5–11.8)	11.3 (10.6–12.0)
No.	6,601	1,000	490
**Aged 40–49 y**			
ASCVD risk (95% CI)	3.8 (3.6–4.0)	4.3 (3.9–4.8)	5.4 (4.5–6.2)
No.	1,944	343	135
**Aged 50–59 y**			
ASCVD risk (95% CI)	8.0 (7.7–8.2)	10.5 (9.4–11.6)	9.8 (8.7–11.0)
No.	1,812	291	179
**Aged 60–69 y**			
ASCVD risk (95% CI)	15.8 (15.3–16.3)	17.4 (16.1–18.6)	16.2 (15.0–17.5)
No.	1,808	261	129
**Aged 70–79 y**			
ASCVD risk (95% CI)	25.5 (25.1–25.9)	24.4 (22.8–26.0)	27.2 (25.5–28.9)
No.	1,037	105	47

Abbreviation: ASCVD, atherosclerotic cardiovascular disease.

a Depression was measured using the Patient Health Questionnaire-9 (PHQ-9). Depression is categorized as not depressed (score, 0–4), mild depression (score, 5–9) and major depression (score, ≥10). All estimates adjust for centered age and centered age squared. Estimates examining all people aged 40–79 additionally include a cubed centered age term.

**Table 3:** Regression Coefficients Showing Association Between Mild and Major Depression with 10-Year ASCVD Risk in People Aged 40–79 y, by Age and Sex, National Health and Nutrition Examination Survey, 2005–2018

Characteristic	Depression^a^				*P* value trend
	Mild	*P* value	Major	*P* value	
**Women, age, y**					
All ages	0.91 (0.64 to 1.19)	<.001	1.65 (1.27 to 2.02)	<.001	.002
40–49	0.39 (0.12 to 0.67)	.006	0.96 (0.57 to 1.35)	<.001	.03
50–59	1.05 (0.54 to 1.56)	<.001	1.41 (0.90 to 1.93)	<.001	.33
60–69	1.53 (0.80 to 2.26)	<.001	2.69 (1.70 to 3.69)	<.001	.04
70–79	0.68 (−0.37 to 1.72)	.20	3.18 (1.61 to 4.75)	<.001	.009
**Men, age, y**					
All ages	1.23 (0.63 to 1.83)	<.001	1.39 (0.72 to 2.07)	<.001	.70
40–49	0.51 (0.02 to 1.00)	.04	1.53 (0.65 to 2.41)	.001	.05
50–59	2.49 (1.40 to 3.58)	<.001	1.83 (0.71 to 2.94)	.002	.42
60–69	1.62 (0.19 to 3.05)	.03	0.45 (−0.96 to 1.85)	.53	.20
70–79	−1.12 (−2.69 to 0.45)	.16	1.67 (−0.05 to 3.39)	.06	.02

Abbreviation: ASCVD, atherosclerotic cardiovascular disease.

a Depression was measured using the Patient Health Questionnaire-9 (PHQ-9). Depression is categorized as not depressed (score, 0–4), mild depression (score, 5–9) and major depression (score, ≥10). To improve model specification, all models include centered age and centered age squared. Estimates for ages 40–79 additionally include a cubed centered age term. Data are from the 2005–2018 National Health and Nutrition Examination Surveys. Coefficients represent the increase in absolute 10-year ASCVD risk relative to the no depression group.

### Lifetime CVD risk

Relative to people without symptoms of depression, people with mild or major depression had higher prevalence of high lifetime CVD risk (Table 4). This finding was observed for both men and women aged 20 to 39 and within each sex-specific 10-year age group. For women aged 20 to 39, high lifetime CVD risk prevalence was 41.9% (95% CI, 39.8%–44.0%) for not depressed, 53.2% (95% CI, 49.6%–56.8%) for mild depression, and 66.5% (95% CI, 61.4%–71.5%) for major depression. For men aged 20 to 39, high lifetime risk prevalence was 53.3% (95% CI, 51.1%–55.5%) for not depressed, 64.8% (95% CI, 60.7%–68.9%) for mild depression, and 74.4% (95% CI, 68.6%–80.2%) for major depression. These values were very similar without age adjustment (Figure). We present percentages for all 5 lifetime CVD risk categories for ages 20–79, by depression and sex, in Supplemental Figure 1 (available at https://osf.io/wcdxv/). Excluding people with a previous cancer diagnosis did not affect the estimates (data not shown) nor did adjusting for education and race and ethnicity (estimates provided in Supplemental Table 2, available at https://osf.io/wcdxv/).

**Table 4:** Prevalence Ratios Showing Associations Between Mild and Major Depression with High (≥39%) Lifetime CVD Risk in People Aged 20–39 y, By Sex, National Health and Nutrition Examination Survey, 2005–2018

Women	Depression^a^					*P* value trend
	Not depressed	Mild	*P* value	Major	*P* value	
**Women, age, y**						
All ages	1 [Reference]	1.27 (1.17–1.38)	<.001	1.59 (1.43–1.76)	<.001	.001
20–29 y	1 [Reference]	1.22 (1.08–1.38)	.002	1.71 (1.47–1.99)	<.001	<.001
30–39 y	1 [Reference]	1.32 (1.19–1.47)	<.001	1.51 (1.34–1.69)	<.001	.05
**Men, age, y**						
All ages	1 [Reference]	1.22 (1.13–1.30)	<.001	1.40 (1.28–1.52)	<.001	.008
20–29 y	1 [Reference]	1.28 (1.14–1.44)	<.001	1.55 (1.35–1.77)	<.001	.02
30–39 y	1 [Reference]	1.16 (1.07–1.26)	<.001	1.29 (1.16–1.43)	<.001	.15

a Depression was measured using the Patient Health Questionnaire-9 (PHQ-9). Depression is categorized as not depressed (score, 0–4), mild depression (score, 5–9) and major depression (score, ≥10). All models include centered age within the respective age band. Data are from the 2005–2018 National Health and Nutrition Examination Surveys.

**Figure:**
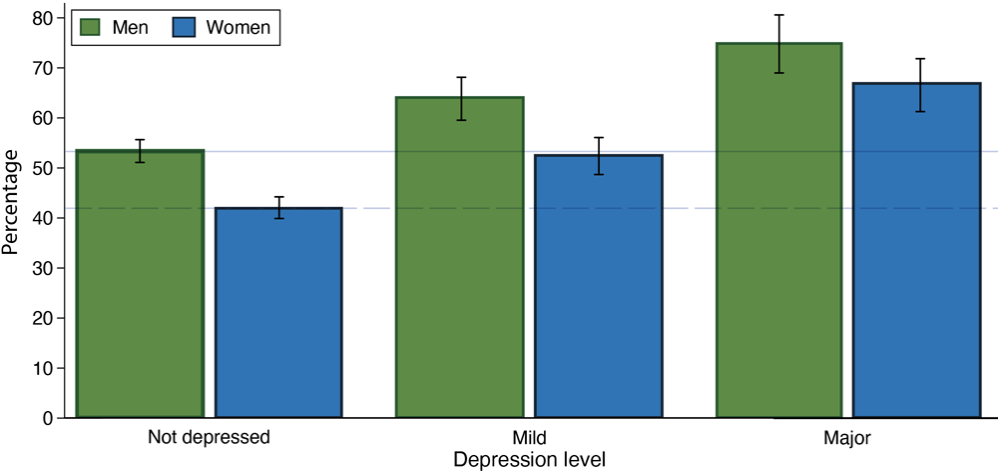
High (≥39%) lifetime cardiovascular disease risk prevalence, by sex and depression status, men and women aged 20 to 39 years, National Health and Nutrition Examination Survey, 2005–2018. Depression was measured by using the Patient Health Questionnaire-9 (PHQ-9). Depression was categorized as not depressed (score, 0–4), mild depression (score 5–9), and major depression (score ≥10). High lifetime risk was categorized with no exclusions for prior CVD (14). Percentages were weighted to represent the subpopulations but are otherwise unadjusted. The solid line is the percentage of high lifetime risk among men aged 20 to 39 years without depression (53.4% [95% CI, 51.1%–55.7%]); the dashed line is the percentage of high lifetime risk among women aged 20 to 39 years without depression (42.0% [95% CI, 39.9%–44.2%]). Error bars represent 95% CIs.

**Table:** 

Depression status	Men	Women
	% (95% CI)	
No depression	53.4 (51.1–55.7)	42.0 (39.9–44.2)
Mild depression	63.9 (59.5–68.1)	52.4 (48.7–56.1)
Major depression	75.2 (69.0–80.6)	66.8 (61.3–71.8)

## Discussion

In a nationally representative US sample of 16,824 people aged 40 to 79 years, we found elevated 10-year ASCVD risk among people with both major and mild depression relative to people without symptoms of depression. This finding is consistent with previous reports that used alternative assessments, such as the Beck Depression Inventory and 10-year Framingham risk scores (15), with studies predicting continuous PHQ-9 depression with ASCVD risk algorithms validated in China (25) and with greater risk of hard CVD events for both clinical and subclinical depression in prospective studies using varied depression assessments (5). We found a similar pattern for high lifetime risk of CVD death among 10,588 people aged 20 to 39 years, where high lifetime CVD risk prevalence was roughly 10% higher for those with mild depressive symptoms relative to people without symptoms of depression, and an additional 10% higher among those with major depression relative to those with mild depression. This finding suggests that despite the advantages of 10-year risk scores, they underestimate the true prevalence of CVD risk among people with mild and major depression.

Although observed absolute 10-year risk differences across depression categories were modest, approximately 23% of NHANES participants aged 40 to 79 had mild or higher depression symptoms in the previous 2 weeks. Therefore, these modest risk differences are likely to generate a substantial number of incident CVD events (26). The public health importance of this association is particularly important for women since the modestly higher ASCVD risk (0.5%–3%) across mild and major depression categories applies to 1 in 4 CVD-free women (approximately 14,002,000 [95% CI 12,938,000–15,066,000]) in the population aged 40 to 79. Similarly, younger adults with mild or moderate depression have clinically relevant elevations in lifetime risk relative to people without depression. This elevated risk is driven solely by modifiable risk factors and supports calls for primary CVD prevention at younger ages (27).

These estimates used standard depression and CVD risk assessments and therefore provide a more practical characterization of modifiable CVD risk by depression, which together contribute substantially to illness and death in the US and worldwide. These population-based benchmarks may be used as a referent for patient registries, clinical practices, and public health surveillance. In turn, widespread adoption of the PHQ-9 and these 2 CVD assessment standards can establish a harmonized research environment providing consistent, comparable data to compare treatment approaches and treatment outcomes and maximize the inferential leverage of these assessments across cohorts and administrative databases (8).

Adult depression screening is recommended in the US, provided that adequate resources exist for treatment and follow-up (6). Adopting the PHQ-9 for screening is recommended (7), and together with common laboratory assessments, these assessment standards can facilitate the clinical detection and management of depression and CVD risk. The ASCVD risk estimates we present are approximately age-independent within the age/sex groups and thus generally reflect modifiable risk factors amenable to primary prevention. Standardized depression assessment also permits linking established benchmarks for changes in depression (eg, remission, defined as a PHQ-9 score decreasing from >9 to <5) (7) to changes in CVD risk.

Ten-year risk differences by depression were weaker among men aged 60 to 79. In this age range, depression is less common and absolute 10-year risk is higher. Thus, the differing base rates for both depression and CVD risk relative to younger cohorts likely contributes to the decoupling of ASCVD risk with depression in older men. The higher absolute ASCVD risk for older men would warrant more aggressive risk factor management irrespective of depression, as would absolute risk among women aged 70 to 79.

Earlier work found a high (approximately 50%) prevalence of high lifetime risk in US adults aged 20 to 39 (20). We found that high lifetime risk is substantially higher with depression, in that 66% of depressed women and 74% of depressed men were at high lifetime risk of CVD death. The association of high lifetime risk with depression in younger adults has to our knowledge not been estimated previously, and this association provides a foundation for CVD risk screening and depression management in younger adults. Lifetime risk differences by depression in younger adults are important because risk factor exposure is cumulative, many people with low 10-year risk have high lifetime risk (28), and depression is associated with elevated CVD risk in even younger people (ie, children and adolescents) (29). Long-term CVD risk assessment is important for younger patients (28), and early life is a critical period for preventing both depression (30) and CVD (27).

The depression–CVD risk association is likely reciprocal, underscoring the advantages of considering them together. For example, people with depression are more likely to smoke, and changes in body mass index (but not insulin levels) portend later depressive episodes, at least in young women (31). Depression is associated with social isolation (32), a marker of premature mortality risk. Biologic pathways, such as cytokines, are implicated in the etiology of depression, but clinical translation of these potential mechanisms is unrealized (33). More broadly, depression is a general CVD risk marker because it predicts pathologically diverse CVD subtypes (34) and is robust to adjustment for many traditional CVD risk factors (4,5). Beyond traditional risk factors, social determinants of health are strongly predictive of depression (35) and CVD (36) and may represent an important “upstream” cause of both conditions.

### Strengths and limitations

Our study had many strengths including a large, diverse probability sample of US adults aged 20 to 79 years. Both depression and CVD risk factors were modeled by using validated approaches common to clinical practice, and we examined both 10-year and lifetime CVD risk. The sensitivity of our analyses to depression levels was maximized by use of a well-defined and homogeneous nondepressed reference group (37). Partitioning mild depression from the typically aggregated subclinical depression category identified 10% to 15% of the population who had elevated CVD risk despite being below depression treatment thresholds. We also addressed statin and antidepressant use, which could potentially modify the observed association. Overall, our study provides population-based estimates of the association of depression with CVD risk using harmonized, widely available depression and CVD risk assessments. These estimates indicated elevated 10-year risk and substantially elevated lifetime CVD risk for clinical and subclinical depression. These data can facilitate primary prevention of CVD risk in the clinic where ASCVD risk scores are the standard, and they spotlight the need to consider lifetime CVD risk when identifying depression in people too young for ASCVD risk assessment.

ASCVD risk scores are sensitive to age and sex, which are not modifiable and thus are limitations of the pooled cohort equations. We attempted to minimize the influence of age on the depression–ASCVD association by examining risk within 10-year age strata and by statistically controlling for age. However, residual age confounding may exist. This limitation does not apply to lifetime CVD risk estimates, which do not include age or sex in the calculations (14). The PHQ-9 cannot distinguish between bipolar and unipolar depression (38) and may be less sensitive to major depression relative to structured clinical interviews. Whether the associations between depression and CVD risk apply to persistent or recurrent depression, or to depression symptom referent periods longer than the past 2 weeks, is unknown. The inability to stratify these estimates by race and ethnicity is another limitation of the study.

These cross-sectional data cannot determine a causal association between depression and CVD risk. Unplanned analyses of small, randomized trials are consistent with a causal association between depression and CVD risk, in that depression treatment reduced 10-year ASCVD risk scores (39) and CVD events (40) among people free of clinical CVD.

### Conclusion

We found that, in a large representative sample of US residents, both mild and major depression were associated with modestly higher absolute 10-year ASCVD risk for people aged 40 to 79 and substantially higher prevalence of high lifetime CVD risk among men and women aged 20 to 39. Primary prevention is optimized by considering both physical and psychological conditions (41), and use of standard, widely available assessments of depression and CVD can advance this important effort.
